# Lecture on Eczema, Including Its Impetiginous, Lichenous, and Pruriginous Varieties

**Published:** 1863-09

**Authors:** T. McCall Anderson

**Affiliations:** Physician to the Dispensary for Skin Diseases; Physician to the Deaf and Dumb Institution, etc.


### LECTURE ON ECZEMA,

### INCLUDING ITS IMPETIGINOUS, LICHENOUS, AND PRURIGINOUS VARIETIES, DELIVERED AT THE DISPENSARY FOR SKIN DISEASES, GLASGOW.

By T. McCALL ANDERSON, M.D., F.F.P.S., Physician to the Dispensary for Skin Diseases; Physician to the Deaf and Dumb Institution, etc.

Reprinted from the "Medical Times and Gazette."

You will remember, gentlemen, that in the preceding lecture, on entering upon the treatment of eczema, I endeavored to impress upon you the necessity, in the first place, of attending to the state of the internal organs, and more particularly to the condition of the stomach and bowels. I then discussed those cases which are under the influence of cod-liver oil, generous diet, iron, and tonics generally, and entered upon the consideration of that class of cases in which the general health appears excellent, and which are more amenable to arsenic, sulphur, and alkalies. The preparations of *sulphur*, though not so generally useful as those of arsenic, are highly serviceable in the treatment of some cases of eczema, especially when the eruption occurs in persons of the lymphatic temperament, and when it is on the decline. When speaking of the administration of purgatives, I told you that the advantage which a sulphur purge possesses over a calomel one is, that it is eliminated in part by the skin, and exercises an alterative effect upon that structure. If, however, we wish to avail ourselves to the full extent of its alterative action, it is advisable to prescribe a course of one of the natural mineral waters which contain sulphur. Those of Harrogate and Moffat in this country, and of Enghien, Barèges, and Luchon on the continent, have the greatest reputation in this respect; and, while some of these waters may be had from the apothecary, it is always more judicious, when it can be effected, to send your patient to the spring itself; for he is thus certain to get the waters fresh and pure, and, away from home and the fatigues and anxieties of business, his body is at the same time invigorated and his mind refreshed.

*Alkalies* are not nearly so generally employed as the preparations of arsenic and sulphur in the treatment of eczema. They are most beneficial when the patient is much addicted to the use of stimulants, and when there is a tendency to acidity of the stomach and to the deposit of lithates in the urine, or to gout or rheumatism. The preparation most in vogue is aqua potassæ, which may be given, largely diluted with water, in doses of twenty minims thrice daily to an adult. The alkali which I am most in the habit of using, however, and which has not, I think, been tried hitherto in this country for such a purpose, is the sesquicarbonate of ammonia in doses gradually increasing from ten up to thirty or even forty grains thrice daily, care being taken that the preparation is fresh and of full strength. A dose of forty grains is often borne well by a patient whose stomach has been gradually accustomed to its reception, while a smaller dose often occasions vomiting in the case of those who have not been in the habit of taking it. Sometimes it is well to continue the ammonia with Fowler's solution or one of the other arsenical preparations. If there is a decidedly gouty tendency, small doses of wine of colchicum, (say ten drops,) and in rheumatic habits, the acetate or bicarbonate of potash (in half-drachm doses,) may be added to each dose. The alkalies must be given *largely diluted* with water, and the dose must be gradually increased till the medicine disagrees or the eruption begins to fade.

*Hydrocotyle Asiatica* has been greatly extolled of late, especially by the French, in the treatment of eczema. It has been very little used in this country, however, although it seems well worthy of a trial, if we may judge from the high encomiums which have been passed upon it by our continental brethren. I have not yet tried it in Dispensary practice, owing to the narrow-minded policy of the French manufacturer, who sells each little bottle of granules at five francs; and, as there are about eighty granules in a bottle, it follows that every ten or fourteen days five francs must be expended, which is too much for the ordinary run of Dispensary patients. In private practice I have tried it several times, and apparently with benefit; but my experience of it is not yet sufficient to enable me to decide upon its merits. I would advise you, however, to try it in cases which resist the ordinary means of cure. The directions for the use of the granules are given in a paper which is enclosed along with each bottle.

Before leaving the internal treatment, let me lay before you four rules which you must carefully attend to in the employment of alterative medicines:—

1st. Let the dose, at first small, be gradually increased till the medicine disagrees, or till the disease begins to yield, and then let it be gradually diminished.

2d. If the medicine disagrees, do not omit it altogether without very good reason, but try it in smaller doses, or in another form, or omit it for a few days till the bad effects have passed off.

3d. To give it a fair trial, it must be continued for a considerable period of time, because in some cases the eruption does not disappear till after it has been administered for many weeks.

4th. Do not permit the patient to give up taking the medicine till some weeks have elapsed since the complete disappearance of the eruption.

If, as I hope, I have convinced you of the great benefit which accrues from the judicious selection of internal remedies in the treatment of eczema, and of their power, in many instances, of removing the eruption when administered alone, you will, perhaps, be hardly prepared for the statement which I make, as the result of some *experience*, that the *local* treatment is even more effectual than the constitutional, although it must be confessed that the applications made use of by many practitioners in this country are unfortunately too often ineffectual, and not unfrequently injurious.

I shall not attempt a description of all the preparations in general use in the local treatment of eczema,—some of them good, some useless, many hurtful,—but shall endeavor to lay before you a short account of those which I have found most valuable, and, what is of the greatest importance, to point out, as far as possible, the indications for their use.

The first point in the local treatment of every eczematous eruption, without exception, is to remove all the crusts which have formed upon it. Till this is done, we can only guess at the condition of the parts beneath; our applications must, in consequence, be selected at random, and these cannot reach the diseased surface whose condition they are intended to modify. You will often meet with opposition on the part of the patient or friends in carrying this injunction into effect, either owing to their laziness, to their preconceived opinions, or to the pain which is sometimes experienced in the removal of the crusts. Patients come here, day after day, informing me that they have done what they could, but have only partially succeeded. You should, in such instances, repeat your instructions, and send your patient home again, for my invariable rule is to refuse to prescribe any local applications till the diseased surface is fully exposed to view, by which means much less time is lost in the end, and the subsequent treatment is much more satisfactory.

The removal of the crusts is a very easy matter, and each practitioner has his own favorite method of procedure. I usually recommend a poultice composed of crumb of bread and hot almond oil to be applied to the eruption at night, and if the crusts do not come away with the poultice in the morning, the part is lubricated with fresh almond oil, and the crusts removed with the finger nail about half-an-hour afterwards, when they have become thoroughly softened. When the crusts reappear, as frequently happens, especially at the commencement of the treatment, they must invariably be removed before the reapplication of the curative agent.

Let us suppose, now, that all the crusts have been removed, and the diseased surface fully exposed to view, what local applications are we to make use of?

If the eruption has just made its appearance, if the surface is acutely inflamed, if it is studded with numerous vesicles or pustules, but particularly if *burning heat* is complained of in place of itching, local sedatives must be employed. A very good application is a cold potato-starch poultice, a small quantity of a powder containing camphor being sprinkled over its surface before being applied, which relieves, at once, the burning heat.

℞. Camphoræ, .................................. ʒss.

Alcoholis, .................................. q. s.

Pulv. Oxydi Zinci, ......................

Pulv. Amyli, .............................. āā ʒiij. M.

*Sig.* Sprinkle a little over the part, or upon the poultice occasionally, to ally the burning heat. Let a small quantity be made at a time, and let the powder be kept in a stoppered bottle, as it loses it strength by exposure to the air.

Or, instead of poultices, emollient ointments may be employed, such as the simple or benzoated oxide of zinc ointment, cucumber ointment (Neligan), or cold cream. A mixture of powdered oxide of zinc and glycerine, in the proportion of half an ounce of the one to two ounces of the other, forms likewise a very soothing application, and to these may be added a little camphor if necessary.

| | | | |
|---|---|---|---|
| ℞. | Camphoræ, | ℈ij. | |
| | Pulv. Oxydi Zinci, | ℥ss. | |
| | Glycerinæ, | ℥ij. | |
| | Cochinillini, | gr. ij. | |
| | Spt. Rosarum, | ʒj. | M. |

*Sig. Stir the mixture before using it.* Rub a thin layer over the inflamed part twice or thrice daily. A most elegant formula.

When the disease becomes chronic, as is indicated more particularly by the disappearance of the burning heat, and the supervention of itching, the local applications which are appropriate are very different, but even they vary according to the stage of the eruption.

If there is *infiltration* of the skin, to any extent, the local treatment which I am in the habit of prescribing is that recommended by some continental dermatologists—in connexion with which the name of Hebra must always be honorably associated, —and which has only of late began to peep forth in fragments in English Medical Journals. This is the treatment by means of *potash* applications, which has been uniformly adopted at the dispensary, and with great success. Having had the privilege, some years ago, of witnessing the carrying out of this means of cure in Hebra's wards at Vienna, some of the prescriptions may resemble very much, or even be identical with, those of that distinguished dermatologist, though I am unable to state at this moment which are due to him and which are mere modifications of my own. I trust, however, I have sufficiently done justice to his merits, and that you will acquit me of the desire of taking any credit, except in so far as the success of this treatment has been first thoroughly established in this country by my colleague, Dr. Buchanan, and myself.

The strength of the local application varies with the amount of the infiltration, and likewise with the extent of the eruption; for, of course, when the disease is extensive, it would be injudicious to make use of those very strong applications which may be applied with safety in the more circumscribed cases.

If the infiltration is slight, or the rash extensive, common *potash soap*, (soft soap, black soap, sapo mollis, sapo viridis,) or a solution of one part of it in two of boiling water, a little oil of rosemary or citronella being added to conceal, in part, the odor, may be used.

| | | | |
|---|---|---|---|
| ℞. | Saponis Mollis, | ℥i. | |
| | Aquæ Bullientis, | ℥ij. | |
| | Olei Citronellæ, | ʒss. | M. |

A piece of flannel dipped in this should be rubbed as firmly as possible over the affected parts night and morning, and the solution allowed to dry upon them, though it should be washed off before each reapplication; or a piece of flannel wrung out of the solution may be applied to the part, and left in contact with it all night if the patient can bear it.

A more elegant preparation is the *aqua potassæ*, (Ed. Ph.,) which may be painted over the eruption night and morning with a large brush, its irritant action being neutralized by means of cold water when the smarting becomes excessive.

Instead of soft soap or aqua potassæ, solutions of *potassa fusa* may be employed. In the mildest cases, with only slight infiltration, two grains of potassa fusa in an ounce of water; in the more severe, five, ten, twenty, thirty grains or even more. An ounce of water may be used, but I rarely resort to a stronger solution where the eruption is extensive. Even the solution containing thirty grains to the ounce, which may be applied in the same way as aqua potassæ, must be washed off with water very speedily, and the application should not be repeated oftener than once daily at the most. When such a strong solution is prescribed, and, especially if the eruption is extensive, it is advisable for the physician to apply it himself, at first at all events. The solution has been used too strong, or been allowed to remain on too long, if it produces any manifest destruction of the skin. When the eruption is very limited and very obstinate, a much stronger solution may be applied, and Hebra sometimes uses a solution of one drachm of potassa fusa in two drachms of water, or even employs the solid caustic itself. This must be done, however, with the greatest circumspection, and the caustic washed off almost immediately, else it is certain to produce great destruction of the skin.

When these strong applications are used, and there is a tendency to the formation of fissures, it will be well to apply cod-liver oil or glycerine to the parts every night, by which means that brittle condition of the skin, which is so much favored by the use of potash locally, and which leads to the formation of fissures, is in part avoided.

Instead of potassa fusa, some recommend solutions of chloride of zinc in similar proportions, but I have very little experience of it, being so well satisfied with the performances of the former. The following case, however, proves that it is a useful agent:—

Hugh D., aged about 40, saddler, came to the Dispensary for Skin Diseases, March 17, 1862. Small patches of eczema were noticed on the backs of his hands, sides of his fingers, and about his wrists. These were very itchy, with a good deal of infiltration; some of them studded with vesicles, and exuding a serous fluid, others dry and scaly. Although some of the patches were situated over the joints of the fingers, there were no fissures. A solution of chloride of zinc (℈j. to the oz. j. of water,) was ordered to be painted over the affected parts morning and evening, and, if the action was too severe, it was to be moderated by the use of cold water. In the intervals between the applications he was to bathe the hands repeatedly in cold water.

March 24.—Greatly improved; itching nearly gone; infiltration of skin much diminished; serous exudation very slight, and only after the application of the zinc lotion.

The patient noticed a slight tendency to the formation of new vesicles on and around the patches, which was at once checked, however, by the lotion.

March 31.—Eruption gone.

When any of these irritants are made use of, they cause smarting, and, when stronger mixtures are applied, often considerable pain; but patients have informed me that, although the smarting and pain are severe, they prefer it to their old enemy, the itching. On the other hand, some patients, although this is rarely the case, will not submit to a repetition of the remedy. I was particularly struck with this in the case of a medical man in this city, who consulted me some time ago, about an extensive eczematous eruption of old standing, and for whom I prescribed the mildest of the applications above referred to. He told a friend shortly after, that he had applied it once, and that it had nearly killed him, the fact being that he had been affected with eczema so long, and had tried so many useless drugs, that his faith in the effect of remedies was shaken, and he would not give a fair trial to a system of treatment which, though a little unpleasant at first, would certainly have cured him. But medical men are notoriously the worst and most refractory patients to deal with.

Having pointed out to you that the strength of the potash or zinc solutions which are employed varies with the amount of infiltration of the skin, it will probably have occurred to you that when the eruption is extensive, and some of the patches much more infiltrated than others, a weak solution may be applied to the latter, a stronger one to the former; and it is equally obvious that, as the infiltration subsides, the solution may be gradually diluted.

Often, by continuing the use of a weak potash solution for some time after the infiltration is gone, all trace of the complaint disappears, but in most instances it is better to substitute for it one of the preparations about to be mentioned as the disease verges upon a cure. But if, on changing the local application, the infiltration of the skin reappears to any extent, you must at once have recourse to the potash solutions again. I have just one caution to give to you before leaving this subject, namely, that you must be careful in the use of these solutions, and especially the stronger of them, in the case of infants, of delicate females, or of old and infirm persons, as the shock produced by their application may possibly be followed by serious results.

While these applications are being employed, cold water forms a very agreeable and useful adjunct. The affected parts may be bathed repeatedly with it during the day, and it is advisable that it should be allowed to fall upon them from a height. Sometimes cloths wrung out of cold water may be placed upon the eruption with advantage in the intervals between the applications, and, if the rash is very extensive, much relief is experienced by plunging into a cold bath, or making use of the shower-bath or cold douche.

I have already pointed out that in mild cases the eruption is often kept up by the scratching alone, and that in these instances local sedatives have sometimes the effect of curing the disease by allaying the itching, and the desire to scratch the part. Hence you will understand how, in more severe cases, while the scratching does not of itself keep up the disease, it certainly tends to aggravate it and to make it more rebellious.

We must therefore exhort the patient to refrain from scratching, as much as possible, and at the same time we must employ means to allay the itching. The potash and zinc preparations have certainly this effect in a marked degree, and so has the application of cold water (for the time); but sedatives and narcotics taken internally are not, in my opinion, of the slightest service, except in so far as a large dose may produce sleep, and, when the patient has long been deprived of it, owing to the itching, this is much to be desired. Lotions of dilute *hydrocyanic acid*, in proportions varying from ♏v. to ʒj. (Ed. Ph.) in an ounce of water or glycerine, may be applied with advantage whenever the part is itching, instead of *giving way to the desire to scratch.*

When such a strong solution as ʒj. of prussic acid to the ℥j. of water is used, it must not be applied over a very extensive surface, and the patient must be warned that it is a very powerful poison. You will often find it of advantage to combine the prussic acid with one of the potash applications formerly referred to, in the proportion say of ♏x. to an ℥j. of the mixture.

℞. Potassæ Fusæ, .............................. gr. x.

Acid, Hydrocyan dil. (Ed. Ph.)........... ℈j.

Aquæ Rosarum,.............................. ℥ij. M.

*Siq.* Rub a little firmly over the eruption night and morning, and when the itching sensation is severe.

Some prefer the use of cyanide of potassium in the form of ointment. For this purpose, from five to ten grains may be mixed with cold cream, or the benzoated oxide of zinc ointment.

℞. Cyanidi Potassi,......................... gr. vj.
Cerati Galeni, (Paris Codex,)......... ℥j.
Cochinillini,.............................. gr. j. M.

*Siq.* Rub a little firmly over the parts which are itching, but let none of the ointment remain undissolved on the skin.

*Tarry preparations* are of the greatest value as local applications in the treatment of eczema, though they are of no use whatever when administered internally. They have long been in vogue in this country, but have too frequently been used in a routine way, and without discrimination. You must, therefore, bear in mind the fact, that they are chiefly of use in the declining stages of eczema, when the infiltration and itching are moderated.

Common tar (*Pix liquida,*) is the application most frequently used at the dispensary, on account of its cheapness, but in private practice you may employ more elegant preparations, such as the oleum rusci or oleum cadini, (oil of cade.) The latter, which is the product of the dry distillation of the wood of the *Juniperus Oxycedrus** is manufactured at Aix-la-Chapelle, and you must be sure that it is got there, else you may have sent to you a liquid prepared from common tar.

Whichever of these preparations you select should be rubbed firmly over the eruption by means of a piece of flannel, and allowed to dry upon it. It should be applied thrice daily, and washed off as well as possible with soft soap, or, amongst the higher classes, with petroleum soap.

Several kinds of soap containing tar and oil of cade, under the name of tar and cade soaps (the latter should be obtained from Aix-la-Chapelle, but is very expensive,—3s. 6d. a cake,) are used by some instead of these preparations in their pure

* "Medicines: their Uses and Mode of Administration," by J. Moore Neligan, M.D. Fourth Edition. Pp. 455. Dublin.

state. If you employ them, you should use them like common soap,—only rub them more firmly over the parts,—and in many cases you will find it of advantage to allow the soap to dry upon the eruption.

I rarely employ tar alone, however, in the treatment of eczema, but usually combine it with one of the potash solutions, in which case you may use it before the infiltration has subsided; for, while I told you that tar was most useful in the declining stages of eczema. I merely meant you to understand that it should not entirely take the place of the potash or zinc solutions while the infiltration was considerable. A most admirable preparation, one of Hebra's, and which is used to a great extent at the dispensary under the name of "tinctura saponis viridis cum pice," and with the most charming effect, is a mixture of equal parts of common tar, methylated spirit, and soft soap, which should be used exactly in the same way, and as frequently as the simple solution of soft soap.

The following case, reported by my assistant, Mr. Arthur Jamieson, many similar to which you have an opportunity of seeing almost every day at the dispensary, illustrates the beneficial effects of this mixture:—

William S., aged 45, laborer, came to the Dispensary for Skin Diseases, on November 10, 1862. He was affected with "eczema impetiginodes" of both ears and the whole of the face, with the exception of the nose. He complained of intense itching, with considerable heat of the parts. The exudation, of a purulent nature, was abundant, and in many parts had concreted, forming large crusts which almost entirely covered the whole of his face. The infiltration of the skin was great; his general health pretty good.

He was ordered a calomel and scammony purge, and a mixture of oxide of zinc and camphor, in the proportion of gr. xx. of the latter to oz. j. of the former, was applied night and morning to the eruption after the removal of the scabs.

13th.—No improvement; ordered the tinctura saponis viridis cum pice.

24th.—Eruption completely gone.

In private practice, where expense is less an object than the elegance of the preparation, you may substitute oil of cade for common tar, and rectified spirit for methylated spirit, while a little oil of lavender may be added to conceal in part the disagreeable odor, or, instead of using soft soap at all, a solution of potassa fusa may be added to the mixture, the amount of the caustic potash depending upon the amount of infiltration of the skin.

℞. Saponis Mollis, ..............................

Spt. Rectificati,..............................

Olei Cadini,.................................. āā ℥j.

Olei Lavandulæ, .............................. ℥jss. M.

*Sig.* Rub a little firmly over the eruption night and morning, and wash it off before each reapplication.

*The preparations of mercury and sulphur*, justly esteemed in the treatment of eczema, are most beneficial when the eruption is verging upon a cure, when the infiltration and exudation are gone, and the itching moderated.

*Of the mercurials*, citrine ointment is my favorite application, though the red and white precipitate ointments are, perhaps, equally useful, or the "Unguentum hydrargyri iodidi" of the London Pharmacopœia. These may be used of full strength or diluted with lard, and, if it is indicated, a few grains of cyanide of potassium may be added to allay the itching. If a lotion is preferred, from one to four grains of the bichloride of mercury may be dissolved with the aid of a little alcohol and mixed with an ounce of rose water, while a little dilute hydrocyanic acid may be added if necessary, the solution being rubbed into the part two or three times daily. In using mercurial preparations locally, you must always bear in mind the possibility of their being absorbed in sufficient quantity to produce salivation; hence you must be careful in anointing an extensive surface, and should warn the patient to discontinue the application if the gums become tender.

It was only the other day that I ordered a lotion of bichloride of mercury (gr. ij. to the ℥j. of water,) to be applied to the nose of a lady, and in three days, to my astonishment, salivation had occurred. On the other hand, I have repeatedly ordered stronger lotions to be applied to extensive surfaces for weeks without the occurrence of the slightest tendency to salivation, thus showing the peculiarities of different constitutions.

*Of the preparations of sulphur*, the common sulphur ointment, of full strength or diluted, and with or without the addition of cyanide of potassium, forms a very useful application. In some cases you may add a little bicarbonate of potash with advantage.

℞. Cyanidi Potassii,......................... gr. v.
Sulphuris,...................................
Bicarbonatis Potassæ,.................... āā. ʒss.
Cochinillini. ................................ gr. i.
Axungiæ, *ad.*,............................... ℥j. M.

A drachm of sulphur mixed with an ounce of alcohol forms a capital lotion, but you must tell the patient to shake the bottle before pouring out the liquid, as the sulphur falls to the bottom. This should be rubbed firmly over the part night and morning and allowed to dry upon it. If the patient is drinking sulphur waters, at Moffat, Harrogate, or elsewhere, and especially if the rash is on the decline, he may combine their external use with their internal administration in the shape of warm baths.

When an ointment is employed in the treatment of eczema, you must give full directions as to the manner of applying it. A very small quantity should be melted on the point of the finger, and rubbed firmly into the affected part, and none of it should be allowed to lie undissolved upon the skin, nor, in most instances, should its color be perceptible after its application; the surface should merely have the appearance of having been recently moistened with pure water. The part should always be cleaned with soap and water before reapplying the ointment; for if you smear layer after layer of it upon the skin, it becomes rancid, acts as an irritant, and is calculated rather to be prejudicial than otherwise.

Astringents are of use in some cases of eczema, such as the sulphate of zinc or copper in proportions varying from three to twenty grains in an ounce of rose-water or solution of the diacetate of lead, diluted with distilled water, but I rarely have occasion to use them, and I think you will find them inferior to the remedies previously described.

For the purpose of curing a very mild, or preventing a threatened attack of eczema, or obviating the occurrence of an immediate relapse, the skin may be washed occasionally with soft soap and water. In private practice, you may recommend the use of Hendrie's "Dispensary Petroleum Soap," which is sold at sixpence per cake, and which is one of the most delightfully perfumed soaps with which I am acquainted.

The following case of eczema erythematodes is of value as illustrative of many of the points of treatment to which I have adverted when the eruption covers an extensive surface:—

A gentleman from the West of Scotland, aged about 40, consulted me on November 9, 1861, about an eczematous eruption of great severity and of many weeks' duration. (He had one previous attack, which lasted three years.) The parts affected were the neck, lower part of the abdomen, inner aspects of the thighs, and the arms and legs, especially on the flexor surfaces of the elbows and knees. The eruption was bright red, and presented an erythematous surface, neither vesicles, pustules, nor papules being visible. There was no exudation from the abdomen or extremities. The skin of the neck, on the other hand, was much infiltrated, and from it serum exuded in abundance. The itching was severe. He was robust, without being corpulent, and, with the exception of the eruption, was in perfectly good health. He was ordered to rub the inflamed parts firmly morning and evening with a piece of flannel dipped in the following mixture:—

| | | |
|---|---|---|
| ℞. | Acidi Hydrocyanici, dil., ................ | ♏xl. |
| | Saponis Viridis, ........................... | ℥iss. |
| | Aquæ, ........................................ | ℥iij. |
| | Olei Rosmarini, ........................... | ʒj. |

Cold water was to be frequently dashed over the parts, five drops of Fowler's solution taken thrice daily after food, and a farinaceous diet recommended.

November 12.—No change. Local application omitted, being too weak. The whole eruption was painted with a solution of potassa fusa, (ʒss. to the ℥j. of water,) which was washed off with cold water whenever the smarting became very severe. This was followed by the exudation of a considerable quantity of serum, especially from the neck. The patient was ordered to repeat this every two or three days, oftener or seldomer according to the severity of the application and the effect produced. The cold shower-bath was to be used twice daily, and the Fowler's solution to be continued.

In a letter, dated November 21, I was informed that the infiltration had quite disappeared from the arms, legs, and abdomen, and only some redness and itching remained. The infiltration, exudation, and itching of neck were much moderated. He was ordered to continue the potassa fusa solution to the neck, and the following mixture was to be rubbed firmly over the other parts night and morning:—

| | | | |
|---|---|---|---|
| ℞. | Acidi Hydrocyanici, dil., ................. | ♏xl. | |
| | Olei Cadini, ................................... | ʒj. | |
| | Saponis Viridis, .............................. | ℥ij | |
| | Olei Rosmarini, ............................. | ʒiss. | |
| | Aquæ, ad., ..................................... | ℥v. | M. |

The Fowler's solution, which agreed, was to be increased to seven and a-half drops thrice daily. The bowels and kidneys being torpid, a teaspoonful of a powder containing sulphur, magnesia, and bitartrate of potash, was to be taken at bedtime.

On December 6, patient stated:—"Since I last wrote, the complaint spread down the legs to the ankles. I have thus been affected from the ear to the foot, first and last. The strong application (potassa fusa, ʒss., aquæ, ℥j.,) checked the inflammation, and no exudation took place." The previous eruption he stated to be rapidly disappearing under the influence of the local applications, although the itching was considerable at times.

On December 30, 1861, only a little roughness and very slight occasional itching of the skin remained. The following ointment was to be applied night and morning:—

℞. Cyanidi Potassi,........................ gr. xii.
Unguenti Oxydi Zinci Benzoati,....
Unguenti Citrini,...................... āā. ℥j. M.

On January 9, 1862, the patient came to see me. The eruption was gone, and there was only a feeling as if the skin was not so elastic as natural. The local treatment was omitted, the dose of the Fowler's solution diminished to five drops thrice daily, and the purgative powder was only to be taken to relieve constipation.

January 1, 1863.—No return of the eruption. Treatment omitted ten months ago.

There can be no doubt that the local treatment was the most effectual in this case.

When the eczematous eruption occupies a limited extent of surface, it usually requires to be attacked by strong local applications, while it is not, as a rule, so much under the influence of internal medicine as when it covers a large area. In such cases, strong solutions of potassa fusa or chloride of zinc, or even these caustics in the solid form, may be employed locally in the manner and with the precautions previously described, and often with benefit; but you must remember to omit them whenever you have removed the infiltration of the skin.

Cauterization with solid nitrate of silver may sometimes be resorted to instead of the above, or the tincture of iodine painted over the part night and morning, and a poultice of bread and hot water applied about once a-week to remove the red skin which forms a covering to the eruption, and prevents the new layers of iodine from coming in contact with the disease itself. But of all the local means for the removal of limited eczematous eruptions, none are equal to blistering them. This may be done by means of a solution of bichloride of mercury, (ʒj. to the oz. j. of alcohol,) the fluid being painted over the eruption, and allowed to dry upon it. The action of the mercurial is, in this case, almost entirely local, and I have never witnessed any effect upon the system at large from its application.

The best blistering agent, however, is the glacial acetum cantharidis,—that is, acetum cantharidis prepared with glacial acetic acid,—the ordinary solution of the Pharmacopœia being too weak. It should be made in small quantities at a time, and kept in good a stoppered bottle, the stopper being removed for as short a time as possible, and, when not in use, covered with leather, otherwise its strength soon diminishes, and much annoyance is thereby occasioned. A little of this solution should be taken up by means of a paint-brush, and painted firmly over the part till it becomes perfectly white. If the fluid is of full strength, and the skin thin, as on the face, it usually blisters it at once; but if the opposite holds, and especially if the head or palms of the hands are to be blistered, it may require to be painted over them for several minutes. After the skin is *thoroughly whitened*, a hot poultice may be applied to make the blister rise. One application is often sufficient to remove the eruption; but, if necessary, it may be repeated weekly, the crust produced by the previous eruption being softened with oil and removed before each reapplication.

A couple of months ago, a gentleman, aged about 35, and otherwise in perfect health, consulted me with regard to an eczematous eruption on the head of twelve years' duration, for which he had been repeatedly shaved, and had consulted many physicians of eminence. Tar had been applied to the scalp systematically for some time, and every conceivable ointment had been used, but without avail. After his hair was removed, I found that the disease corresponded to the form which I described to you under the name of eczema squamosum: it covered the whole head, and, as usually happens in these obstinate cases, was accurately limited to the hairy parts. The scales on the surface were numerous, the itching severe, and, on the crown, front, and sides of the head, the infiltration and redness of the skin was great. I blistered these parts with glacial acetum cantharidis, the fluid requiring to be painted on for some minutes, owing to the thickness of the skin, and ordered the rest of the scalp, which was less severely affected, to be painted with tincture of iodine morning and evening. In a fortnight the iodine was omitted, and when the crusts and scales produced by the iodine and the blistering fluid were removed, the scalp appeared perfectly healthy and without a vestige of the previous eruption. To consolidate the cure, however, tincture of iodine was painted over the whole head night and morning for a fortnight, and, when the red skin was removed, the scalp looked remarkably well, there being not even the vestige of a scale, which can rarely be said even of the head of a healthy person. No other treatment was resorted to, and the gentleman has since been in America. In the interval his hair grew in greater force than ever, and he is delighted to be rid of his old and indefatigable enemy.

Many cases such as these might be mentioned, but I shall just refer to one more, which many of you had an opportunity of seeing:—A woman, pretty well advanced in years, came to the dispensary a few months ago, to get advice about an eczematous eruption of old standing, which covered the whole of the palmar surface of each hand. She had likewise a tendency to eczema of the leg, which was removed by means of the "tinctura saponis viridis cum pice," a preparation previously referred to. It is of the hands, however, I wish to speak. The eruption here assumed that form which I described to you as eczema rimosum, the fissures being very numerous and deep, and the infiltration of the skin great. Itching was mingled with the pain, but the latter, on account of the fissures predominated. Owing to the pain and stiffness, the hands were kept constantly in a semi-closed position, and she was unable to use them. I blistered each hand with the glacial acetum cantharidis, which had a marvellous effect. The eruption disappeared completely, and the patient returned with joy depicted in her countenance, and opened and closed her hands with perfect facility, not unmingled with pride.

